# Ultrasonic-Assisted Extraction of *Codonopsis pilosula* Glucofructan: Optimization, Structure, and Immunoregulatory Activity

**DOI:** 10.3390/nu14050927

**Published:** 2022-02-22

**Authors:** Hai-Yu Ji, Juan Yu, Jian-Shuang Jiao, Xiao-Dan Dong, Sha-Sha Yu, An-Jun Liu

**Affiliations:** 1 College of Biotechnology, Tianjin University of Science and Technology, No. 29, 13th Street, Tianjin Economic Development Area, Tianjin 300457, China; 2 College of Food Science and Engineering, Tianjin University of Science and Technology, No. 29, 13th Street, Tianjin Economic Development Area, Tianjin 300457, China; yujuan14615@tust.edu.cn (J.Y.); 19848945@mail.tust.edu.cn (J.-S.J.); 19946014@mail.tust.edu.cn (X.-D.D.); 20942009@mail.tust.edu.cn (S.-S.Y.)

**Keywords:** *C. pilosula* glucofructan, structural characteristics, immunoregulatory activity

## Abstract

In recent years, multiple edible polysaccharides from *Codonopsis pilosula* were mainly isolated with high average molecular weights and exhibited various bioactivities, but it was proven that low-molecular-weight polysaccharides could exert stronger activities due to the superior water solubility and permeability. In the present study, the water-soluble polysaccharide *C. pilosula* with low molecular weight was isolated under ultrasonic assistance at 30 °C, the extraction process was optimized via response surface method (RSM), and the structure and immunoregulatory activity were further investigated. The maximum yield (4.86%) for crude polysaccharides (cCPPs) was obtained under following parameters: ultrasonic power of 370 W, liquid/material ratio of 33 mL/g, ultrasonic time of 81 min. Subsequently, the cCPPs were further purified through dialysis and Sephadex G-25 column to acquire purified polysaccharide (CPPs). Structural analysis indicated that CPPs was a glucofructan (average molecular weight of 4.23 × 10^3^ Da) with (2→1)-β-D-Fru*f* and (1→)-α-D-Glc*p* as the backbone branched by (2→6)-β-D-Fru*f*. Additionally, CPPs could enhance immunoregulatory function by stimulating NO production and cytokine (IL-6 and TNF-α) secretion of RAW264.7 macrophages dose-dependently, which presented no cytotoxic effects. These data suggest that CPPs have the potential to be used as a nutritional dietary compound and natural immunostimulant supplement in the food industry.

## 1. Introduction

Recently, herbaceous plants have become an increasing research focus because of the potential application prospects in food, medicine, and cosmetics fields. *Codonopsis pilosula* Nnannf (belonging to the bellflower family) is a well-known edible and functional herbaceous perennial mainly located in northern regions of China, and has been usually served as a ginseng alternative (more expensive) due to the similar biological features, including effectively strengthening health, invigorating the spleen, and nourishing the lungs [1,2]. The polysaccharides are identified as the major effective constituents of *C. pilosula* and exhibit multiple biological functions, including immunomodulatory [3], antivirus [4], and anticancer activities [5]. However, diverse extraction methods would contribute to different extraction yields of polysaccharides, as well as the structural properties and biological effects [6]. Therefore, more gentle extraction technology for polysaccharides needs to be explored, with the aim of maintaining the physicochemical and functional properties.

Classical extraction methods for polysaccharides usually require elevated temperatures and a long period of time, which might cause the degradation and denaturation of the structure, leading to weakened bioactivities [7,8]. Presently, various modern extractive techniques have been applied to commercially extract functional biopolymers with a high extraction yield and low depletion of resources under moderate conditions [9]. Ultrasound treatment could produce acoustic cavitation bubbles in water solution, and the bubbles’ collapse would accelerate substance dissolution via disrupting cell walls in a eco-friendly green environment [10], contributing to the incremental extraction yields with curtate processing time and less energy consumption [11,12]. As reported, ultrasonic extraction had been extensively used in the preparation of multiple bioactive polysaccharides to improve the extraction yields [13]. Response surface methodology (RSM) could evaluate the impacts of processing parameters on response values by planning experiments and constructing models [14], and has been applied for optimizing the extraction processes of bioactive compounds for higher yields [15]. Therefore, in this study, crude *C. pilosula* polysaccharides (cCPPs) were prepared at a moderate temperature (30 °C) under ultrasonic extraction, and the extraction conditions were optimized by RSM, which would be beneficial to efficient utilization and industrial production.

The body immune responses (including innate and adaptive responses) are elicited by various environmental antigens and orchestrated by multiple immune cells [16], which are essential for immunodeficiency-related diseases [17]. Macrophages mainly exist in tissues or peripheral blood, and play a central role in host defense by removing pathogens and dead cells [18,19]. Therefore, the in vitro evaluation of macrophages activities after bioactive substance exposure is an important indicator to reflect the immunoregulatory capacity of compounds [20]. As reported, the molecular weights of polysaccharides would significantly affect the solubility and bioactivities, and low-molecular-weight polysaccharides could exhibit stronger activities due to the higher dissolubility and permeability compared with those of higher molecular weight [21]. As a consequence, purified *C. pilosula* polysaccharides (CPPs) with low molecular weight were prepared through dialysis and column chromatography, and the immunostimulatory effects on macrophages were evaluated.

In the present study, cCPPs were extracted from edible roots of *C. pilosula* under an ultrasonic-assistant process at 30 °C, and the extraction conditions were optimized to improves yields using RSM on the basis of single-factor experiments. Subsequently, the CPPs with low molecular weight were isolated and the structural properties were analyzed. Finally, the immunoregulatory action on macrophages of the CPPs were investigated. These results would promote the practical applications of CPPs as an edible compound in the food industry and help to improve the nutritional values of *C. pilosula*.

## 2. Materials and Methods

### 2.1. Raw Materials and Reagents

The roots of *C. pilosula* Nnannf from Pingshun County of Changzhi city (Shanxi Province, China) were purchased through Tianjin Taijin Technology Co., Ltd. (Tianjin, China). Bovine serum albumin (BSA), phosphate buffered saline (PBS), and 3-(4,5-dimethyl-2-thiazolyl)-2,5-diphenyl-2-H-tetrazolium bromide (MTT) were provided by Solarbio Biological Technology Co., Ltd. (Beijing, China). T-series dextran and neutral red were provided by Sigma-Aldrich Co. (St. Louis, MO, USA). A nitric oxide (NO) assay kit was purchased from Beyotime Biotechnology Co., Ltd. (Shanghai, China), as well as a Mouse TNF-α ELISA Kit and Mouse Interleukin 6 (IL-6) ELISA Kit from ZCIBIO Technology Co., Ltd. (Shanghai, China). All other chemicals were of analytical grade.

### 2.2. Preparation Process of cCPP

The root powder of *C. pilosula* was immersed and extracted with distilled water at 30 °C using a DXIENTZ-IID ultrasonic homogenizer twice. The collected leach liquor was merged, concentrated, and treated by 3 volumes of absolute ethyl alcohol at 4 °C overnight. The sediments were dissolved in distilled water and treated by the Sevag reagent to obtain cCPP. The yield of cCPP was calculated following the formula below:

Yield of cCPP (%) = (cCPP weights/*C. pilosula* roots powder weights) × 100

### 2.3. Single-Factor Experiments

Four crucial factors, including the liquid–solid ratios (10–50 mL/g), ultrasonic power (200–520 W), ultrasonic time (20–100 min), and extraction times (1–5 times), were optimized in single-factor experiments for cCPP preparation. In each single-parameter experiment, one contributing element was researched and the other parameters were constant. The unchanged values were identified as having a liquid–material ratio of 20 mL/g, ultrasonic power of 360 W, ultrasonic time of 60 min, and 2 extraction times. Each experiment was put into practice three times.

### 2.4. Experimental Design and RSM Modeling

The extraction conditions for cCPP were further optimized by Box–Behnken design (BBD) of the RSM according to the above results. Three operating parameters, including liquid–material ratio (A, 25−35 mL/g), ultrasonic time (B, 70−90 min), and ultrasonic power (C, 320−400 W), were coded at three levels of −1, 0, and +1, respectively, which are shown in Table 1.

The 17 experimental combinations were run with random sequence, and the predicted and actual yields are displayed in Table 2. As presented, the extraction yields of obtained cCPP in the actual experiments were close to those of the predicted values, suggesting a well-fitted model with high precision [21].

### 2.5. Purification of CPPs

The prepared cCPPs under the above optimal conditions were dialyzed with a molecular weight cut off (MWCO) of 1000 Da against tap and distilled water for three days and centrifuged (8000 rpm for 10 min) to remove sediment. The polysaccharide solution was further purified by sephadex-G25 column. Finally, the purified *C. pilosula* polysaccharides (CPPs) were obtained after collection and lyophilization.

### 2.6. Primary Characterization of CPPs

#### 2.6.1. Chemical Composition Detection

The contents of total sugar, protein, uronic acid, and total polyphenols in the CPPs were examined through the phenol-sulfate colorimetry method, Coomassie brilliant blue protein assay [22,23], carbazole-sulfate method [24], and the Folin–Ciocalteu method, respectively [25].

#### 2.6.2. Molecular Weight Determination of CPPs

The molecular weight of the CPPs was evaluated by high-performance gel permeation chromatography (HPGPC) (Agilent-1200 series, Santa Clara, CA, USA) equipping a TSK-gel G4000PWxL (7.8 mm × 300 mm), and T-series dextran with molecular weights of 110 kDa (T110), 70 kDa (T70), 40 kDa (T40), 10 kDa (T10), and 3 kDa (T3) was employed as standard.

#### 2.6.3. Monosaccharide Constituents of CPPs

The monosaccharide compositions and the contents of the CPPs were detected using high-performance liquid chromatography (HPLC, Agilent-1200, USA) equipped with an amino column and refractive index detector. In short, 5 mg of dried CPPs were treated by 2 M trifluoroacetic acid at 100 °C for 1 h. The products were neutralized by NaOH solution, followed by HPLC detection. The determination parameters were set as below: The column and detector temperature was 35 °C, the injection volume was 10 μL, the flow rate was 1.0 mL/min, and the mobile phase was acetonitrile–water (7:3). In addition, the fructose content was also further confirmed according to the Seliwanoff colorimetric method.

#### 2.6.4. Nuclear Magnetic Resonance (NMR) Determination of CPPs

Dried CPPs (approximately 40 mg) were dissolved in 0.5 mL deuteroxide (99.9%, D_2_O) and transferred to an NMR tube for ^1^H, ^13^C, COSY, HSQC, and HMBC NMR spectra determination using an Advance DPX-400 NMR spectrometer (400 MHz) (Bruker, German).

### 2.7. Immunoregulatory Activity of CPPs

#### 2.7.1. Cell Viability Detection

An MTT assay was utilized to evaluate the viability of murine macrophages (RAW264.7 cells) after CPP treatments. Briefly, 20,000 RAW264.7 cells per well were planted into a 96-well plate, cultured at 37 °C for 24 h, and then treated with various concentrations of CPPs (6.25–400 μg/mL) for 20 h. Lipopolysaccharide (LPS) in a concentration of 1 μg/mL was regarded as positive control, and the isovolumetric medium as negative control. Subsequently, an MTT solution (5 mg/mL) of 10 μL/well was inoculated and cultured for another 4 h. The medium was abandoned, followed by a 150 μL DMSO addition into each well to dissolve the formed blue-purple formazans. Finally, the solution could be measured at 490 nm using a microplate spectrophotometer (Synergy HTX, Bio Tek, Winooski, VT, USA).

#### 2.7.2. Phagocytosis Detection by Neutral Red Uptake

Neutral red reagent was applied to assess the phagocytosis abilities of RAW264.7 cells. RAW264.7 cells in a concentration of 20,000 cells/well were inoculated in a 96-well plate and cultured under an atmosphere (5% CO_2_, 37 °C) for 24 h. LPS in a concentration of 1 μg/mL was regarded as positive control, and the isovolumetric medium as negative control. Then CPPs (6.25–400 μg/mL) were added for 24 h incubation, and 100 μL of 0.075% neutral red solution was added to each well for another 1 h incubation. Subsequently, the superfluous neutral red was removed by washing three times with PBS, and 200 μL of cell lysis solution containing 1% acetic acid-anhydrous alcohol (1:1, *v:v*) was added to each well to release the neutral red from macrophages at room for 2 h. The absorbance at 540 nm was measured and the cell phagocytic uptake rates were calculated [26].

#### 2.7.3. NO, TNF-α, and IL-6 Level Detection

The nitric oxide (NO) assay kit was employed to evaluate the NO levels in the medium supernatant. About 400,000 RAW264.7 cells were seeded into each well of a 24-well plate for 24 h cultivation and then treated with different concentrations (6.25–400 μg/mL) of CPPs for 24 h. LPS in a concentration of 1 μg/mL was regarded as positive control, and the isovolumetric medium as negative control. After incubation, the supernatant of each well was collected separately, and the NO level was determined according to the kit instruction. The absorbance (540 nm) was measured under the microplate spectrophotometer, and the NO levels were calculated by establishing a standard curve of different sodium nitrite concentrations. Similarly, the TNF-α and IL-6 levels produced by RAW264.7 cells were evaluated through enzyme-linked immunosorbent assay (ELISA) kits following the corresponding manufacturer’s instructions.

### 2.8. Statistical Analysis

All values are displayed as the mean ± standard deviation (S.D.), and each experiment was repeated three times. The significance of between-group differences was measured by Student’s *t*-test and one-way analysis of variance (ANOVA). A *p*-value of <0.05 was identified as significant.

## 3. Results and Discussion

### 3.1. Single-Factor Experiment Analysis

**Figure 1:**
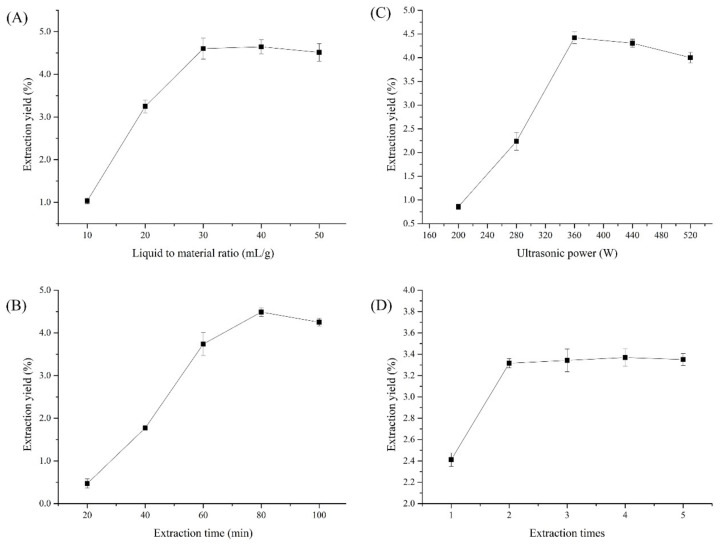
Single-factor experimental results for cCPP extraction, (**A**) Liquid to material ratio; (**B**) Extraction time; (**C**) Ultrasonic power; (**D**) Extraction times.

Figure 1 presents the preliminary optimized results of the extraction conditions via single-factor experiments. As displayed in Figure 1A, the cCPP yields significantly increased with an increasing liquid-to-material ratio from 10 to 30 mL/g, and then gradually stabilized, which is consistent with previous related research [27]. As reported, the improved liquid-to-material ratio could have been beneficial for polysaccharide dissolution, resulting in higher polysaccharide yields until reaching the saturated polysaccharide dissolution [28]. Therefore, a liquid-to-material ratio of 30 mL/g was chosen in subsequent optimizations.

Figure 1B shows the influence of the extraction time (20–100 min) on cCPP yields. The extraction time also demonstrated a significant impact on polysaccharide yields, which gradually increased to maximum as the extraction time extended to 80 min, and then also slightly decreased [29]. This phenomenon may also have resulted from the degradation of the cCPPs, which was similar with ultrasonic power. Thus, an extraction time of 80 min was selected for subsequent experiments.

Then the effects of ultrasonic power on cCPP yields were investigated, ranging from 200 to 520 W, and the results are presented in Figure 1C. As displayed, the cCPP yields rapidly increased as ultrasonic power improved from 200 to 360 W, and then gradually decreased when the ultrasonic power was higher than 360 W, which was attributed to the polysaccharide degradation under superior powers, which is in agreement with a related report [30]. Therefore, an ultrasonic power of 360 W was adopted in subsequent BBD experiments.

The effects of extraction time (1, 2, 3, 4, 5) on cCPP yields were investigated, and the results are presented in Figure 1D. The cCPP yields were remarkably improved as the extraction frequency increased from 1 to 2, and then gradually became a stable value, which is consistent with a previous paper [31]. As a result, the extraction frequency of 2 was selected for cCPP preparation.

### 3.2. ANOVA for cCPP Extraction

Design-Expert software (version 10.0) was used to evaluate the ANOVA for the response of the cCPP extraction, and the results of the ANOVA for the quadratic model are displayed in Table 3. The experimental variables were associated with the following formula:

Yields = 4.68 + 0.23*A* + 0.12*B* + 0.63*C* − 0.025*AB* − 0.15*AC* − 0.13*BC* − 0.18*A*^2^ − 0.47*B*^2^ − 1.33*C*^2^

The variance analysis demonstrated that coefficients *A*, *B*, *C*, *AC*, *BC*, *A*^2^, *B*^2^, and *C*^2^ were extremely significant (*p* < 0.01), whereas the interaction effects of *AB* showed no significant difference on cCPP yields, which indicates that the variables *A*, *B*, *C*, *AC*, *BC*, *A*^2^, *B*^2^, and *C*^2^ could significantly influence the extraction yields of cCPPs. As shown, the *p* < 0.0001 of this mode and the *p* = 0.7258 for the lack-of-fit value indicate insignificant effects of deviation and suggest a fitted model [32]. Besides, the difference of less than 0.2 between the Pred *R^2^* and Adj *R^2^* values also indicates a good correlation between the predicted and actual cCPP extraction yields [33]. 

### 3.3. Interaction Effects of Every Two Parameters and Verification Experiment

**Figure 2:**
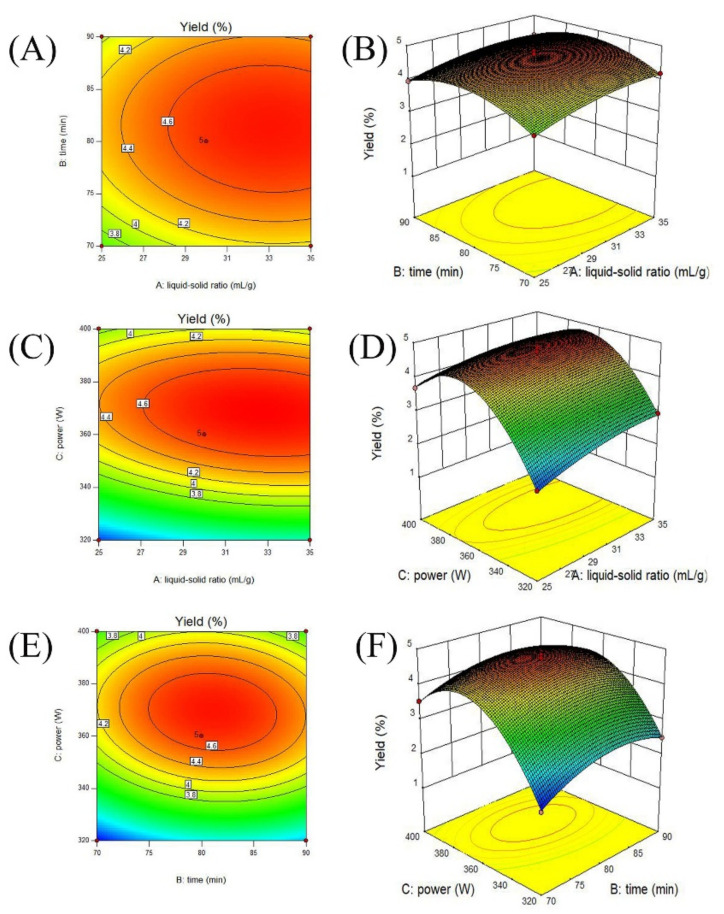
Contour plots (**A**,**C**,**E**) and response surface (**B**,**D**,**F**) of the polysaccharide production with various variables, including liquid-to-material ratio, ultrasonic time, and ultrasonic power.

Figure 2 presents the interaction effects of the liquid-to-material ratio (*A*, 25–35 mL/g), ultrasonic time (*B*, 70–90 min), and ultrasonic power (*C*, 320–400 W) on cCPP yields. As shown, all response surfaces curves exhibited a maximum point in these selected experimental ranges, suggesting a reasonable range of factors. As reported, the elliptical/circular contour plots reflected significant/indistinctive interaction effects between these variables [34]. Therefore, the liquid-to-material ratio and ultrasonic power (*AC*), ultrasonic time, and ultrasonic power (*BC*) exhibited obvious interaction effects (*p* < 0.01) on the cCPP extraction yields, which is consistent with previous results.

The optimal extraction conditions for maximum cCPP yields were determined by Design-Expert software: liquid-to-material ratio of 32.81 mL/g, ultrasonic time of 80.86 min, and ultrasonic power of 368.02 W, with a predicted yield of 4.81%. These conditions were slightly modified for more convenience and feasibility in practical production (liquid-to-material ratio of 33 mL/g, ultrasonic time of 81 min, and ultrasonic power of 370 W), and the obtained extraction yields of the cCPPs (three parallel trials) reached 4.86 ± 0.16%. The optimized extraction parameters not only helped remarkably improve the extraction yields of the cCPPs, but also effectively avoided the waste of energy and resources, which could also provide material foundation for further polysaccharides purification and bioactivity determination.

### 3.4. Chemical Constitutions and Primary Structure Analysis of CPPs

In this study, the cCPPs were purified through dialysis and a sephadex-G25 column to obtain CPPs with a purification yield of 31.79%. Chemical constitution analysis showed that the sugar and protein contents of the CPPs were 95.64 ± 4.29% and 1.85 ± 0.11%, respectively. In addition, the uronic acid and total phenolic contents were 0.89 ± 0.07% and 1.13 ± 0.12%, respectively, indicating that the CPPs were a kind of neutral polysaccharide.

**Figure 3:**
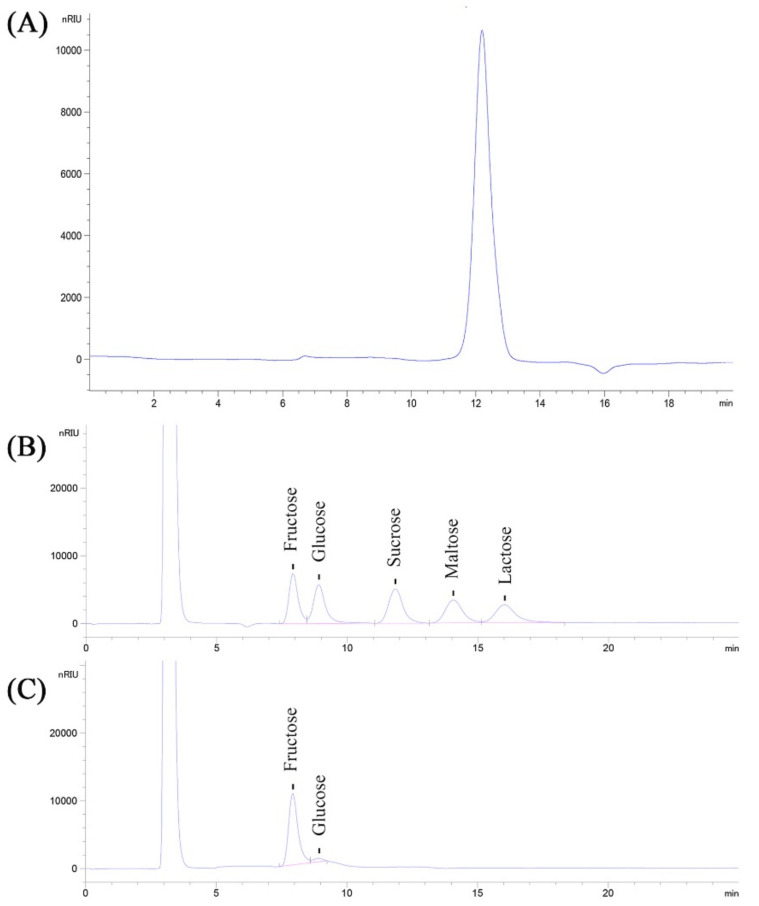
HPGPC (**A**) spectrum and HPLC analysis of standards (**B**) and CPPs (**C**).

The average molecular weight (Mw) of the CPPs was estimated, and the results are shown in Figure 3A. As presented, the retention time (R_t_) of the CPPs in the HPGPC spectrum was 12.196 min, demonstrating that the average molecular weight of the CPPs was about 4.23 × 10^3^ Da, combining the calibration curve of T-series dextran, taking R_t_ as the x-coordinate and lg(Mw) as the y-coordinate.

The monosaccharide compositions and the relative contents were determined by HPLC analysis. As presented in Figure 3B,C, the HPLC spectrum showed two main peaks, which were confirmed to be fructose and glucose, compared to the standards. Moreover, the corresponding contents of fructose and glucose in the CPPs were calculated as 93.14 ± 4.57% and 6.86 ± 0.36%, respectively, according to the calibration curves drawn by various concentrations of standards.

As reported, the structural properties of polysaccharides could significantly affect various properties, including bioactivities, and it has been proven that low-molecular-weight polysaccharides can exhibit superior bioactive effects due to the good water solubility and permeability [35]. Up to now, the average molecular weights of most polysaccharide isolated from *C. pilosula* were usually higher than 5.00 × 10^3^ Da [36], indicating a relatively weak bioactivity presentation. Therefore, in this study, CPPs with low molecular weight were prepared, and the specific structural characteristics and immunomodulatory activity in vitro were investigated.

### 3.5. NMR Spectra Analysis of CPPs

**Figure 4:**
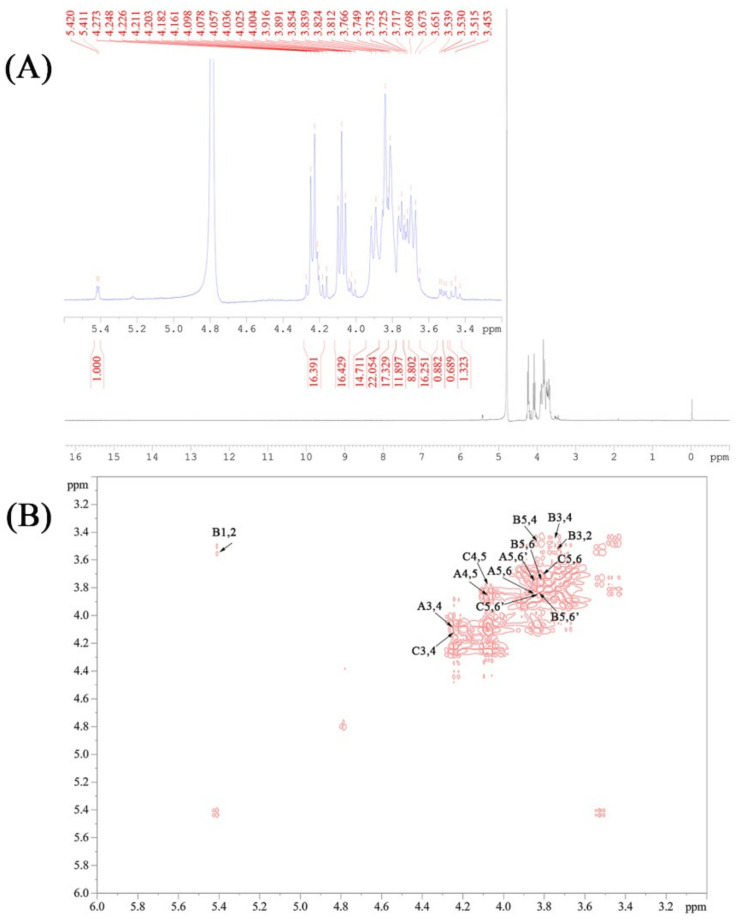
The ^1^H (**A**) and COSY (**B**) NMR spectra of the CPPs.

The signals of ^1^H and COSY NMR spectra were characterized and are presented in Figure 4A,B. As shown, the chemical shifts in the CPPs occurred from 5.50 ppm to 3.45 ppm in ^1^H NMR spectra, and there were no signals between 6 and 8 ppm, indicating that no phenol or ferulic acid existed in the CPPs. According to the ^1^H and COSY NMR analysis, the anomeric protons signals at about 5.45 ppm were attributed to the H1 presence of α-Glc [37]. The H signals at about 4.27 ppm and 4.12 ppm were assigned to Fru-H3 and Fru-H4, respectively. The signals from 3.48 ppm to 3.92 ppm corresponded to Fru-H1, Fru-H5, Fru-H6, and Glc-H2−H6, which were definitely assigned through the cross-peaks of adjacent hydrogens in the COSY spectrum, and would be helpful for further carbon attribution [38]. Additionally, fructose is a ketose sugar, suggesting that the C2 of fructose is anomeric carbon without linked hydrogen. Thus, the cross-absorption signals involved in Fru-H2 are nonexistent in the COSY spectrum.

**Figure 5:**
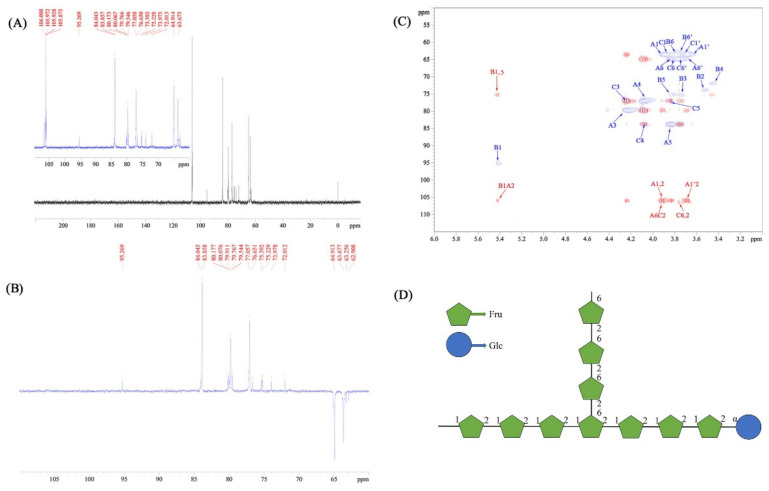
The ^13^C (**A**), DEPT135 (**B**), HSQC/HMBC (blue/red signals) (**C**), and NMR spectra and possible structural formula (**D**) of the CPPs. Note: A represents (2→1)-β-D-Fru*f* residue, B represents (1→)-α-D-Glc*p* residue, C represents (2→6)-β-D-Fru*f* residue.

In terms of the ^13^C NMR spectrum in Figure 5A, the signals of the CPPs appeared from 106.50 ppm to 62.90 ppm. On the basis of the DEPT135 spectrum in Figure 5B, the carbon signals at 106.02 ppm/106.48 ppm were determined to be one quaternary carbon atom; the carbon signals at 79.77 ppm/76.92 ppm, 77.06 ppm/84.04 ppm, and 83.86 ppm/76.66 ppm were confirmed to be the tertiary carbons; and the carbon signals at 63.68 ppm/62.91 ppm and 64.92 ppm/63.26 ppm were the secondary carbons, which means the structures of the A and C units were furan fructose residues with a β glycosidic bond configuration [39]. Figure 5C displays the results of the HSQC (blue signals) and HMBC (red signals) NMR spectra of the CPPs. Combined with the COSY spectrum analysis, the chemical shifts in the carbons were attributed accurately in the HSQC spectrum [40], and β-Fru and α-Glc were marked as A/C and B, respectively, which is shown in Table 4. Based on the above assignments of the ^1^H and ^13^C signals, six cross-peaks, including A1,2 (A1′,2), A6C2, C6,2, B1A2, and B1,5, were observed in the HMBC spectrum. It can be inferred that CPPs mainly contained (2→1)-β-D-Fru*f* and (1→)-α-D-Glc*p* as the backbone, which is analogous to the structure of inulin [41]. Additionally, the branch of (2→6)-β-D-Fru*f* was demonstrated according to the HSQC and HMBC spectra analysis. In view of the above results, the CPPs can be identified as a glucofructan, and the possible structural formula is shown in Figure 5D, which is quite different from the other inulin-type fructan isolated from *C. pilosula* in previous literatures [42,43]. These diversities were probably caused mainly by diverse extraction and purification methods. As reported, the branched degree presented crucial roles affecting various bioactivities of polysaccharides [44]. Compared with the structure of inulin, the CPPs presented the branch of (2→6)-β-D-Fru*f*, which might have been responsible for the activity development.

### 3.6. Immunoregulatory Activity of the CPPs

#### 3.6.1. Cell Viability Determination

**Figure 6:**
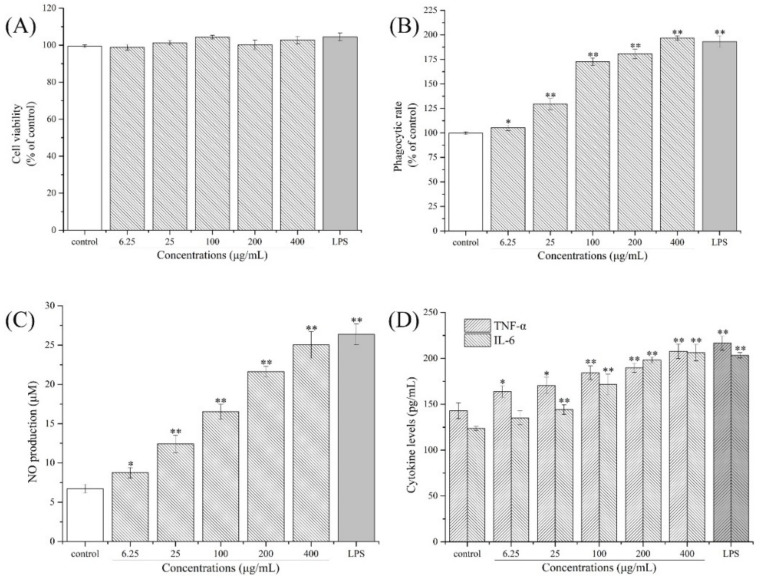
Immunoregulatory effects of the CPPs on Raw264.7 macrophages ((**A**) cytotoxic evaluation, (**B**) phagocytic rates, (**C**) NO production, (**D**) TNF-α and IL-6 cytokines secretion). Significant differences are designated as * *p* < 0.05 and ** *p* < 0.01, compared with the control group.

The effects of the CPPs on RAW264.7 cell viability were determined using an MTT assay, which was a critical indicator for further application, and the determination results are shown in Figure 6A. As presented, the CPPs exhibited no significant cytotoxicity on macrophages even at a concentration of 400 μg/mL. The viabilities of RAW264.7 cells after various concentrations of CPP (6.25, 25, 100, 200, 400 μg/mL) treatments were 98.80 ± 1.54%, 101.29 ± 1.26%, 104.31 ± 2.31%, 100.22 ± 2.51%, and 102.81 ± 1.96%, respectively, suggesting that CPPs would not directly induce any side effects in RAW264.7 cells and could be used as a novel effective immunostimulant. As reported, multiple polysaccharides could not directly exert cytotoxic effects on immune cells, and could be employed for immune cell activation [45], which is consistent with our results.

#### 3.6.2. Macrophage Phagocytosis Analysis

In the present research, the effects of the CPPs on RAW264.7 cell phagocytosis were determined, and the results are presented in Figure 6B. As shown, compared to the control group, the phagocytic rates of RAW264.7 cells after different concentrations of CPP (6.25–400 μg/mL) treatments for 24 h were significantly enhanced by 1.05, 1.30, 1.73, 1.81, and 1.97 times those of the control, indicating that the CPPs could activate RAW264.7 cells and promote the phagocytic capacities. As is known to us, the phagocytic ability is a crucial indicator to evaluate macrophage activities, which would further reflect the immunoregulatory capacity of macrophages after bioactive compound activation [46]. In this study, the edible CPPs exerted an immune-modulating function by enhancing the phagocytic ability of macrophages, which presented similar effects with a neutral polysaccharide from ginger [47].

#### 3.6.3. NO, TNF-α, and IL-6 Content Analysis

The effects of the CPPs on NO, IL-6, and TNF-α production of macrophages were determined and the results are shown in Figure 6C,D. As displayed, CPP treatments of 6.25 μg/mL to 400 μg/mL remarkably improved NO production and TNF-α and IL-6 secretion compared to the control group, dose dependently (*p* < 0.05 or *p* < 0.01). When the CPP concentration reached 400 μg/mL, the contents of NO, TNF-α, and IL-6 increased to 25.03 ± 1.70 μM, 207.77 ± 7.91 pg/mL, and 206.16 ± 9.04 pg/mL, respectively. As reported, NO, IL-6, and TNF-α are secreted by activated macrophages under bioactive compound stimulation, contributing to eliminating pathogenic microorganisms [48]. In general, the NO was first released in the proinflammatory response of the macrophages, and then the cytokines including TNF-α and IL-6 were synthesized to destroy abnormal cells or activate T-helper cells [49,50]. These results suggest that the CPPs could enhance macrophage phagocytosis by improving the NO, TNF-α, and IL-6 levels, demonstrating that the CPPs have the potential to be a novel edible compound from *C. pilosula* to improve the immunoregulatory activity of the body.

## 4. Conclusions

In summary, *C. pilosula* polysaccharides (cCPPs) were separated from *C. pilosula* through ultrasonic-assisted extraction, and the maximum yields of the cCPPs reached 4.86 ± 0.16% under the obtained optimal conditions, including a liquid-to-material ratio of 33 mL/g, ultrasonic time of 81 min, and ultrasonic power of 370 W, which was close to the predicted value (4.81%). Subsequently, the novel low-molecular-weight glucofructan (CPPs) was isolated through dialysis and sephadex G-25 column purification, which presented an average molecular weight of 4.23 × 10^3^ Da with (2→1)-β-D-Fru*f* and (1→)-α-D-Glc*p* as the backbone and (2→6)-β-D-Fru*f* as branches. Meanwhile, the CPPs exhibited strong immunostimulation activity on RAW264.7 cells, which make them valuable for further application in the functional food industry as a novel edible compound.

## Figures and Tables

**Table 1:** Levels and independent variables in BBD matrix.

Independent Variables	Codes	Coded Levels		
		−1	0	+1
Liquid–material ratio (mL/g)	A	25	30	35
Ultrasonic time (min)	B	70	80	90
Ultrasonic power (W)	C	320	360	400

**Table 2:** Predicted and actual extraction yields of cCPP.

No.	Liquid–Material Ratio	Ultrasonic Time	Ultrasonic Power	Extraction Yields (%)	
	(mL/g)	(min)	(W)	Predicted Value	Actual Value
1	−1 (25)	+1 (90)	0 (360)	3.95	3.94
2	0 (30)	0 (80)	0 (360)	4.68	4.67
3	−1 (25)	−1 (70)	0 (360)	3.65	3.66
4	0 (30)	0 (80)	0 (360)	4.68	4.74
5	0 (30)	+1 (90)	−1 (320)	2.50	2.49
6	0 (30)	+1 (90)	+1 (400)	3.49	3.52
7	−1 (25)	0 (80)	+1 (400)	3.71	3.69
8	+1 (35)	0 (80)	+1 (400)	3.88	3.86
9	0 (30)	0 (80)	0 (360)	4.68	4.64
10	+1 (35)	−1 (70)	0 (360)	4.16	4.17
11	0 (30)	−1 (70)	−1 (320)	2.00	1.97
12	0 (30)	0 (80)	0 (360)	4.68	4.62
13	+1 (35)	0 (80)	−1 (320)	2.92	2.94
14	0 (30)	0 (80)	0 (360)	4.68	4.73
15	−1 (25)	0 (80)	−1 (320)	2.17	2.19
16	0 (30)	−1 (70)	+1 (400)	3.51	3.53
17	+1 (35)	+1 (90)	0 (360)	4.36	4.35

**Table 3:** ANOVA for the quadratic model.

Source	Sum of Squares	DF	Mean Square	F Value	*p*-Value Prob > F	Significance
Model	12.86	9	1.43	652.87	<0.0001	Significant
*A*: liquid–solid ratio	0.42	1	0.42	193.31	<0.0001	**
*B*: time	0.12	1	0.12	53.72	0.0002	**
*C*: power	3.14	1	3.14	1433.12	<0.0001	**
*AB*	2.50 × 10^−3^	1	2.50 × 10^−3^	1.14	0.3207	
*AC*	0.084	1	0.084	38.41	0.0004	**
*BC*	0.070	1	0.070	32.08	0.0008	**
*A* ^2^	0.13	1	0.13	61.45	0.0001	**
*B* ^2^	0.94	1	0.94	427.11	<0.0001	**
*C* ^2^	7.46	1	7.46	3408.42	<0.0001	**
Residual	0.015	7	2.19 × 10^−3^			
Lack of fit	3.93 × 10^−3^	3	1.31 × 10^−3^	0.46	0.7258	Not significant
Pure error	0.011	4	2.85 × 10^−3^			
Cor total	12.88	16				

Note: **, *p* < 0.01 represents extremely significant difference.

**Table 4:** The chemical shifts in the CPPs in the HSQC spectrum.

NO. of C/H	A: (2→1)-β-D-Fru*f*		B: (1→)-α-D-Glc*p*		C: (2→6)-β-D-Fru*f*	
	δ_H_	δ_C_	δ_H_	δ_C_	δ_H_	δ_C_
1	3.70/3.89	63.68	5.42	95.27	3.72/3.86	63.49
2	-	106.05	3.53	73.97	-	106.48
3	4.23	79.77	3.76	75.39	4.27	79.55
4	4.08	77.06	3.45	72.05	4.02	76.65
5	3.84	83.86	3.83	76.65	3.83	84.04
6	3.75/3.84	64.91	3.80/3.74	62.70	3.77/3.80	65.04

## Data Availability

Data sharing, not applicable. No new data were created or analyzed in this study. Data sharing is not applicable to this article.
